# Influence of a Diester Glucocorticoid Spray on the Cortisol Level and the CCR4^+^ CD4^+^ Lymphocytes in Dogs with Atopic Dermatitis: Open Study

**DOI:** 10.1155/2014/492735

**Published:** 2014-09-21

**Authors:** Masato Fujimura, Hironobu Ishimaru

**Affiliations:** Fujimura Animal Allergy Hospital, Aomatanihigashi 5-10-26, Minou-shi, Osaka 562-0022, Japan

## Abstract

This study investigated the influence of 0.00584% hydrocortisone aceponate spray (HCA; Cortavance Virbac SA, Carros, France) on blood serum cortisol levels and peripheral blood CCR4^+^ CD4^+^ T-lymphocyte levels in dogs with atopic dermatitis. Patients were randomly divided into group I (*N* = 8) and group II (*N* = 8). The dogs in group I were sprayed with HCA on the affected skin once a day for three weeks. The dogs in group II were treated once a day for 3 days followed by no treatment for 4 days for a total of three weeks. For the dogs in group I and group II the CADESI-03 scores before and after use of HCA showed significant reduction (*P* < 0.01). The postcortisol level after the use of HCA in group I showed 36.0% decrease and showed significant suppression (*P* < 0.01). By comparison, the use of HCA on group II did not show decrease in postcortisol levels. There was a tendency of suppression for hypothalamus—pituitary gland—adrenal gland system, but it was not serious influence. In addition, there was no influence on peripheral blood CCR4^+^ CD4^+^ lymphocytes percentage in dogs in group I after treatment with HCA.

## 1. Introduction

Canine atopic dermatitis is an intractable chronic skin disease, and management requires a combination of many treatments [1]. Treatment generally involves the use of an antifungal drug, an antibiotic, a shampoo therapy, a humidity retention treatment, an anti-inflammatory drug, and/or hyposensitization. The most frequently used therapy is a corticosteroid. Unfortunately there are not many practical guidelines for use of steroid therapy [2] in veterinary medicine, and there are only a few reports about the side effects. The prolongation of steroid treatment can cause atrophy of the skin, ulceration, hair loss, and skin calcification [3], but the adverse reaction we are concerned about most is the influence on hypothalamus—pituitary gland—adrenal gland system [4, 5].

0.00584% hydrocortisone aceponate spray (HCA; Cortavance Virbac　SA, Carros, France) is different from a conventional steroid, due to the fact that the absence of a halogen at C6, C9, and C21 is associated with better local and systemic tolerance [6, 7]. Double esterification of C21 and C17 enhances penetration of the stratum corneum and ensures specific metabolism in the lower dermis. These actions minimize the effects on hair follicles, dermal fibroblasts, and blood vessels, decreasing the likelihood of local cutaneous and systemic adverse effects [6, 7].

The effectiveness of HCA for treatment of inflammation is proved in former studies [8–10]. The purpose of this study is to confirm the influence of HCA on the patient by measuring the clinical score, the cortisol levels by the ACTH stimulation test, and the CCR4^+^ CD4^+^ lymphocytes percentage in blood both before and after use of HCA in randomized clinical study.

## 2. Materials and Methods

### 2.1. Animals

All dogs were diagnosed with canine atopic dermatitis and had severe itch; they were seen at one hospital (Fujimura Animal Allergy Hospital, Osaka, Japan). The study was conducted from June to November, 2011, with the owners' consent.

### 2.2. Diagnosis of CAD (Canine Atopic Dermatitis)

The diagnosis of CAD was made by ruling out other causes of the itch. All dogs received flea control and appropriate treatment for scabies mites. If bacterial pyoderma and yeast (*Malassezia dermatitis*) were diagnosed by cytology, it was treated mainly by shampoo therapy. All dogs underwent an elimination diet using “hypoallergenic” foods (Hill's prescription diet z/d Canine Ultra: Hill's Pet Nutrition, Topeka, KS, USA; or Royal Canin Veterinary Diet Sensitivity Control: Royal Canin, Aimargue, France; or Iams Veterinary Formulas FP: Cincinnati, Ohio, USA) for at least 8 weeks. The diagnosis of CAD was based on compatible history and clinical signs of Favrot's criteria [11]. Intradermal allergy testing was performed with 24 selected antigens (Greer Pharmacy, Lenoir, NC, USA; or Torii Pharmaceutical Co., Ltd., Tokyo, Japan).

### 2.3. Test Method and Cases

Sixteen dogs diagnosed with CAD were distributed into two groups randomly. In group I, four of the eight dogs had positive reactions to house dust mite mix (*Dermatophagoides farinae and D. pteronyssinus*) and the other four showed no reactions to intradermal test and were diagnosed as having atopic like dermatitis. In group II, five dogs had positive reactions to house dust mite mix and the other three were diagnosed with atopic like dermatitis. Owners were instructed to apply to the dog two pumps of HCA spray per 100 cm^2^ of affected skin. HCA spray was mainly applied to axilla and inguina, not to the face. They were instructed not to spray more than 1/3 of the whole body. The clinical evaluation and blood tests were performed on day 0 and day 21. Owners of dogs in group I sprayed HCA on the affected skin once a day for three weeks. Owners of dogs in group II sprayed HCA on the affected skin (all owners use a 7-day cycle of 3 days of spray plus 4 days of no spray, for a total of 3 weeks). Owners were instructed to apply the spray once daily to affected skin only from 10 cm away at a dose rate of two sprays per 100 cm^2^ of affected skin [8].

### 2.4. CADESI-03: Canine Atopic Dermatitis Extent and Severity Index

CADESI-03 was used to assess lesion severity. The severity of erythema, lichenification, excoriations, and alopecia was assessed at 62 body sites using a scale from 0–5 (0 = none, 1 = mild, 2-3 = moderate, and 4-5 = severe) [12]. The same investigator performed all assessments on open labelled study.

### 2.5. Blood Biochemical Test

Blood sample was collected from the jugular vein, and the peripheral blood was used for each inspection. The following inspection was performed before and after use of HCA.

#### 2.5.1. Cortisol Level by the ACTH Stimulation Examination

Cortrosyn (tetracosactide acetate, 0.25 mg/2 mL, Daiichi Sankyo, Tokyo) 5 ug/kg was injected intravenously [13]. The serum was collected before and 1 hour after injection; cortisol level was measured using fluorescence polarization immunoassay (the CLEIA method) in commercial laboratory (Doubutu kensa, Osaka).

#### 2.5.2. Blood Immunologic Test (CCR4^+^ CD4^+^ Lymphocytes: CCR4^+^ Cells in Peripheral CD4^+^ T-Lymphocytes)

A sample of peripheral blood was collected in EDTA before and after use of HCA. The sample was saved by refrigeration and transported to a commercial laboratory (Animal Allergy Clinical Laboratories, Inc., Kanagawa). The CCR4^+^ CD4^+^ T lymphocytes were detected by flow cytometry following methods previously reported [14, 15].

### 2.6. Statistical Analysis　(Wilcoxon Signed-Rank Test)

Statistical analysis was performed by paired *t*-tests and Wilcoxon signed-rank test. Statistical significance was defined as *P* < 0.05.

## 3. Results

### 3.1. CADESI-03: Canine Atopic Dermatitis Extent and Severity Index

In group I, the mean and SD of the total CADESI-03 score before and after the use of HCA was 294.8 ± 175.0 and 208.8 ± 138.4, respectively. A total score rate of decrease was 29.2%, and it was significantly different after use of HCA (*P* < 0.01). In group II, the mean and SD of the total CADESI-03 score before and after the use of HCA was 393.3 ± 171.4 and 335.4 ± 157.0, respectively. A total score rate of decrease was 14.8%, and it was significantly different after use of HCA (*P* < 0.01). Even though, CADESI-03 scores of group I and II are different, both groups showed decrease in the score which were significantly different. It represents the efficacy of HCA on both treatment methods.

### 3.2. Cortisol Level by the ACTH Stimulation Examination

**Figure 1:**
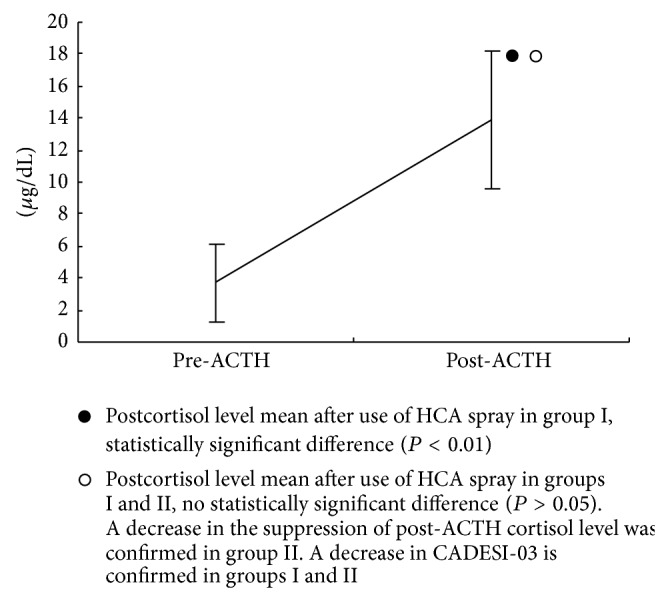
Three weeks use of HCA spray on dogs with CAD, group I (*n* = 8), cortisol level of pre-ACTH and post-ACTH before use of HCA spray, pre-ACTH cortisol level mean ± SD; 3.7 ± 2.4 *μ*g/dL and post-ACTH cortisol level mean ± SD; 13.9 ± 4.3 *μ*g/dL.

**Figure 2:**
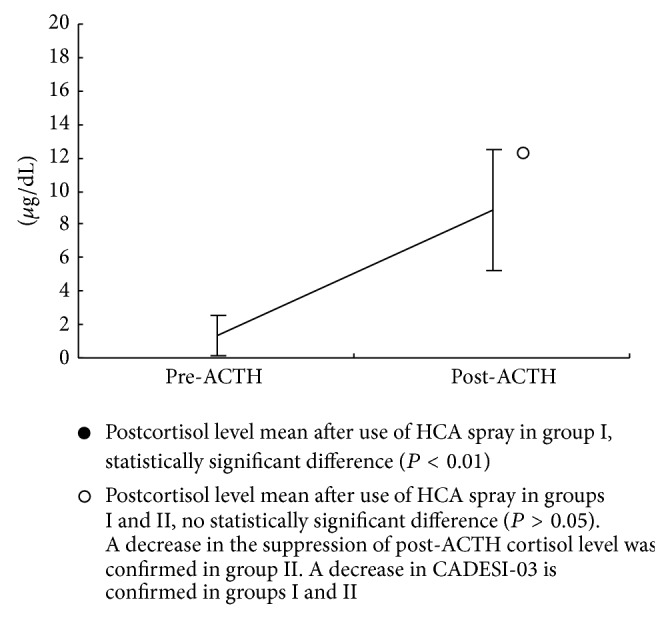
Group I (*n* = 8), cortisol level pre-ACTH and post-ACTH after use of HCA spray for 21 days, pre-ACTH cortisol level mean ± SD; 1.4 ± 1.2 *μ*g/dL and post-ACTH cortisol level mean ± SD; 8.9 ± 3.6 *μ*g/dL.

In group I, prior to the use of HCA, the mean and SD of the cortisol levels were 3.7 ± 2.4 *μ*g/dL (standard range 1.0–7.7 *μ*g/dL) pre-ACTH and 13.9 ± 4.3 *μ*g/dL (standard range 1.0–18.0 *μ*g/dL) post-ACTH, Figure 1. The mean and SD of cortisol levels after use of HCA were 1.4 ± 1.2 *μ*g/dL pre-ACTH and 8.9 ± 3.6 *μ*g/dL post-ACTH, Figure 2.

**Figure 3:**
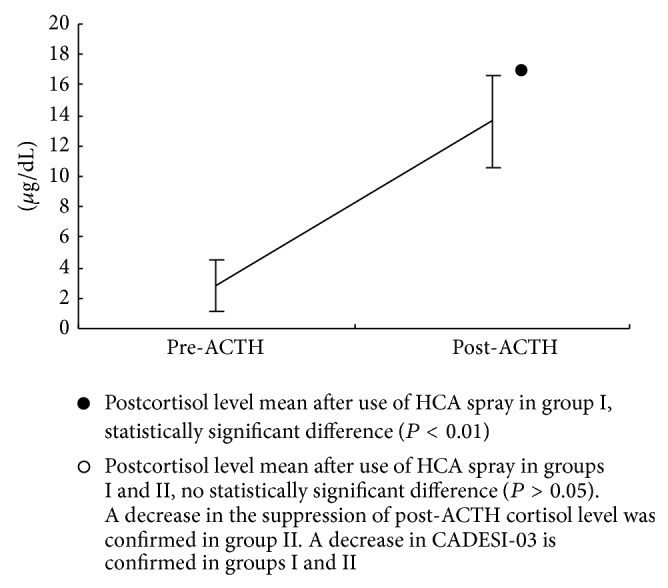
Group II (*n* = 8), cortisol level pre-ACTH and post-ACTH after use of HCA spray for 21 days (use days-limited cases), pre-ACTH cortisol level mean ± SD; 2.8 ± 1.7 *μ*g/dL and post-ACTH cortisol level mean ± SD; 13.6 ± 3.0 *μ*g/dL.

In group II, the mean and SD of cortisol levels after use of HCA were 2.8 ± 1.7 *μ*g/dL pre-ACTH and 13.6 ± 3.0 *μ*g/dL post-ACTH, Figure 3.

In group I, the decrease in cortisol levels post-ACTH before and after use of HCA was 36.0% and showed significant difference (*P* < 0.01). The post-ACTH cortisol level before use of HCA in group I and the postcortisol level after use of HCA in group II showed no significant difference (*P* > 0.05). From these results, limitation use of the spray might recover the postcortisol value of after use of the spray.

### 3.3. Blood Immunologic Test (CCR4^+^ CD4^+^ Lymphocytes: CCR4^+^ Cells in Peripheral CD4^+^ T-Lymphocytes)

**Figure 4:**
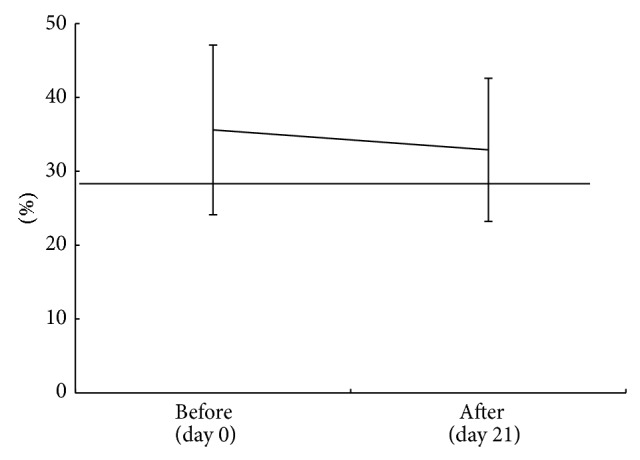
Three weeks use of HCA spray on dogs with CAD, CCR4^+^CD4^+^ lymphocytes percentage before and after use of HCA spray for 21 days, before (day 0) CCR4^+^CD4^+^ mean ± SD; 37.8 ± 10.8% and after (day 21) CCR4^+^CD4^+^ mean ± SD; 39.6 ± 14.4%. Statistically no significant difference was found (*P* > 0.05), Crossbar; <28.7 cut-off value.

In group I, the mean and SD of CCR4^+^ CD4^+^ lymphocytes percentage before the use of HCA was 37.8 ± 10.8%. After the use of the spray the percentage was 39.6 ± 14.4%, Figure 4. CCR4^+^ CD4^+^ lymphocytes percentage did not fall below 28.7%, a cut-off level [15]. The CCR4^+^ CD4^+^ lymphocytes percentage before and after use of HCA did not show significant difference (*P* > 0.05).

## 4. Discussion

After 21 days of HCA use, the total score of CADESI-03 decreased by 29.2% in group I and 14.8% in group II. Dogs in both groups showed significant difference after use of the spray. It represents an effectiveness of HCA which resembles a result in the report of Nutall and others [10], which showed 57.1% decrease in the CADESI-03 on day 28. Our results show a difference in the percent decrease but the reason was unclear.

One report of the use HCA described the skin atrophy as an adverse effect [9]. In the report of Bizikova and others, they confirm mild skin atrophy to be visible in eight of ten dogs in the axillary and inguinal region at the end of the second week of the spray application [9]. Such reduction in dermal thickness was associated with atrophy of dermal collagen and partial atrophy of adnexa with decreased numbers of hair follicles in the anagen phase of the hair cycle. Hair loss was identified in one dog out of the 16 dogs treated. Atrophy was not observed in the report of Nutall and others. It is inspected that the effect of HCA is equal to oral cyclosporine [10]. The advantage of the use of HCA in comparison to the use of oral or parenteral steroids may avoid some systemic adverse effect suppression of the hypothalamic-pituitary-adrenal axis [4, 5] which can be seen occasionally with the administration of systemic steroids. In the report of Bizikova and others [9], the HCA suppressed histamine related intradermal test reactivity distal to the site of application seven days after use of HCA once daily. These data suggest HCA might be absorbed systemically from the skin surface causing anti-inflammatory effect on other remote nontreated regions. Another hypothesis is that the dog might lick sprayed region creating systemic administration which would cause anti-inflammatory effect on the other side of the skin. It was very unique, and this report was the study that understood enough the characteristic of this drug, but it was supplemented by the lack of data. Therefore, in this study, blood cortisol level and CCR4^+^ CD4^+^ lymphocytes percentage were measured to make up for these lacks. This study showed 36.0% decrease in postcortisol value compared with before and after use of HCA and it showed significant difference (<0.01). On the other hand, CCR4^+^ CD4^+^ lymphocytes percentage did not move and showed no effect to it. These results suggest weak suppression of the hypothalamus pituitary gland; adrenal gland axis might be caused by use of HCA. These data support the first hypothesis of Bizikova and others, which is that HCA might be absorbed from the treated skin with activity and it can be said that there was not enough degradation of HCA. However, influence was in the tolerated level, and it can be avoided by limited use of the spray. And there was little influence of the recurrence of the clinical score even if it reduce the frequency of use.

Comparing with the result of past study, use of the HCA showed little influence than other dosage routes [16–20]. For example, the injection of the steroid dosage, oral, showed more suppression to cortisol than that of the spray. In this study, it showed no influence on peripheral blood CCR4^+^ CD4^+^ lymphocytes percentage in group I.

Because a Langerhans cell present antigen to a T-cell in the skin epidermis, these T-cells migrate the main route of the expose to the live body of the sensitization antigen of the CAD to lesion skin. In other words peripheral blood CCR4^+^ CD4^+^ lymphocytes percentage detects the migration degree of these T-cells and it can also judge the allergic strength of the live body or an immunoreactive state [13, 14]. There was no significant difference in CCR4^+^ CD4^+^ lymphocytes percentage before and after the use of HCA in this study. It meant that HCA shows extremely little influence to systemic immunoreaction. The Bizikova and others [9] also performed skin biopsy 24 hours after intradermal test challenge, and intraepidermal histologic measurement of the treatment side showed reduction in the total leukocyte, eosinophile, and CD3^+^ lymphocytes. From two studies, HCA spray infiltrated very small amount systemically, but the main area of the effect is limited to the skin epidermis and restrained inflammation and the cell-mediated immunity in that part, so the influence on systemic immunoreaction might be extremely little.

## 5. Conclusion

HCA spray is a promising next generation anti-inflammatory steroidal therapeutic drug. The use of HCA spray should be compared with other steroid ointments and sprays. It is thought that the use of HCA spray is likely to help the transition from the use of an oral steroid, helping to minimize the development of iatrogenic Cushing disease.

## References

[1] Olivry T., DeBoer D. J., Favrot C. (2010). Treatment of canine atopic dermatitis: 2010 clinical practice guidelines from the International Task Force on Canine Atopic Dermatitis. *Veterinary Dermatology*.

[2] Angus J. C. Canine pruritus-practical guide to management.

[3] Nakamura M., Kawamura Y., Minegishi M., Momoi Y., Iwasaki T. (2004). Hypercalcemia in a dog with resolution of iatrogenic Cushing’s syndrome. *Journal of Veterinary Medical Science*.

[4] Ridgway H. B., Moriello K. A. (1993). Iatrogenic Cushing's syndrome in a dog from owner's topical corticosteroid. *Archives of Dermatology*.

[5] Komiyama N., Tsumagari S., Ohba S., Takagi K., Satoh S., Takeishi M. (1991). Hypophyseal-adrenocortical function in experimental iatrogenic canine Cushing's syndrome. *The Journal of Veterinary Medical Science*.

[6] Brazzini B., Pimpinelli N. (2002). New and established topical corticosteroids in dermatology: clinical pharmacology and therapeutic use. *American Journal of Clinical Dermatology*.

[7] Schackert C., Korting H. C., Schäfer-Korting M. (2000). Qualitative and quantitative assessment of the benefit-risk ratio of medium potency topical corticosteroids in vitro and in vivo: characterisation of drugs with an increased benefit-risk ratio. *BioDrugs*.

[8] Nuttall T., Mueller R., Bensignor E. (2009). Efficacy of a 0.0584% hydrocortisone aceponate spray in the management of canine atopic dermatitis: a randomised, double blind, placebo-controlled trial. *Veterinary Dermatology*.

[9] Bizikova P., Linder K. E., Paps J., Olivry T. (2010). Effect of a novel topical diester glucocorticoid spray on immediate- and late-phase cutaneous allergic reactions in Maltese-beagle atopic dogs: a placebo-controlled study. *Veterinary Dermatology*.

[10] Nuttall T. J., McEwan N. A., Bensignor E., Cornegliani L., Löwenstein C., Rème C. A. (2009). Comparable efficacy of a topical 0.0584% hydrocortisone aceponate spray and oral ciclosporin in treating canine atopic dermatitis. *Veterinary Dermatology*.

[11] Favrot C., Steffan J., Seewald W., Picco F. (2010). A prospective study on the clinical features of chronic canine atopic dermatitis and its diagnosis. *Veterinary Dermatology*.

[12] Olivry T., Marsella R., Iwasaki T. (2007). Validation of CADESI-03, a severity scale for clinical trials enrolling dogs with atopic dermatitis. *Veterinary Dermatology*.

[13] Feldman E. C., Nelson R. W. (2003). *Canine and Feline Endocrinology and Reproduction*.

[14] Maeda S., Ohmori K., Yasuda N. (2004). Increase of CC chemokine receptor 4-positive cells in the peripheral CD4^+^ cells in dogs with atopic dermatitis or experimentally sensitized to Japanese cedar pollen. *Clinical & Experimental Allergy*.

[15] Yasuda N., Masuda K., Maeda S. (2008). CC chemokine receptor 4-positive CD4^+^ lymphocytes in peripheral blood increases during maturation in healthy beagles. *Journal of Veterinary Medical Science*.

[16] Chastain C. B., Graham C. L. (1979). Adrenocortical suppression in dogs on daily and alternate day prednisone administration. *American Journal of Veterinary Research*.

[17] Moore G. E., Hoenig M. (1992). Duration of pituitary and adrenocortical suppression after long-term administration of anti-inflammatory doses of prednisone in dogs. *American Journal of Veterinary Research*.

[18] Kemppainen R. J., Lorenz M. D., Thompson F. N. (1982). Adrenocortical suppression in the dog given a single intramuscular dose of prednisone or triamcinolone acetonide. *The American Journal of Veterinary Research*.

[19] Reeder C. J., Griffin C. E., Polissar N. L., Neradilek B., Armstrong R. D. (2008). Comparative adrenocortical suppression in dogs with otitis externa following topical otic administration of four different glucocorticoid-containing medications. *Veterinary Therapeutics*.

[20] Zenoble R. D., Kemppainen R. J. (1987). Adrenocortical suppression by topically applied corticosteroids in healthy dogs. *Journal of the American Veterinary Medical Association*.

