# Corrigendum to “Influence of a Diester Glucocorticoid Spray on the Cortisol Level and the CCR4^+^ CD4^+^ Lymphocytes in Dogs with Atopic Dermatitis: Open Study”

**DOI:** 10.1155/2016/6510347

**Published:** 2016-01-20

**Authors:** Masato Fujimura, Hironobu Ishimaru

**Affiliations:** Fujimura Animal Allergy Hospital, Aomatanihigashi 5-10-26, Minou-shi, Osaka 562-0022, Japan

Our paper titled “Influence of a Diester Glucocorticoid Spray on the Cortisol Level and the CCR4^+^ CD4^+^ Lymphocytes in Dogs with Atopic Dermatitis: Open Study” [1] contains an error in Abstract and Introduction. The concentration of hydrocortisone aceponate spray was incorrectly reported as 0.00584% while the correct value is 0.0584%.
